# A combination of clinical, electrocardiographic, and echocardiographic parameters predicts pulmonary hypertension occurrence in patients with end-stage renal disease

**DOI:** 10.3389/fcvm.2024.1337243

**Published:** 2024-11-18

**Authors:** Handong Ding, Fei Zhang, Jinbiao Zhong, Jiashan Pan, Yiding Chen, Ji Zhang, Qin Wang, Guiyi Liao, Zongyao Hao

**Affiliations:** ^1^ Department of Urology, The First Affiliated Hospital of Anhui Medical University, Hefei, China; ^2^ Institute of Urology, Anhui Medical University, Hefei, China; ^3^ Anhui Province Key Laboratory of Urological and Andrological Diseases Research and Medical Transformation, Hefei, China; ^4^ Pharmacy Department, The First Affiliated Hospital of Anhui Medical University, Hefei, China

**Keywords:** electrocardiography, echocardiography, pericardial effusion, pulmonary hypertension, end-stage renal disease

## Abstract

**Background:**

Pulmonary hypertension (PH) in patients with end-stage renal disease (ESRD) has a high incidence rate and mortality and its early identification is critical. However, whether a combination of clinical, electrocardiographic, and echocardiographic parameters can predict the occurrence of PH in patients with ESRD remains to be elucidated. Herein, we evaluated the predictive value of the combined score of these parameters.

**Methods:**

Data from 370 patients with newly diagnosed ESRD who underwent routine echocardiography and electrocardiography between May 2016 and May 2017 were retrospectively evaluated. The incidence of PH during a 60-month follow-up period was investigated. Twenty-one patients were excluded due to incomplete data among other reasons. Finally, 349 patients were included in the analysis, of whom, 158 (45%) developed PH.

**Results:**

Analysis of electrocardiogram reports suggested that a corrected Q-T interval (QTc) of >438.5 ms was associated with PH. Echocardiographic reports suggest that left atrial diameter (LAD), interventricular septum thickness in end-diastole (IVSd), stroke volume (SV), and pericardial effusion are also associated with PH development. Results of multivariate Cox analysis showed that LAD >3.785 cm, IVSd >1.165 cm, SV >79.5 ml, QTc >438.5 ms, and pericardial effusion were independent predictors of PH in patients with ESRD. The incidence of new-onset PH increased significantly with increasing composite scores, that is, the sum of risk scores determined using hazard ratios.

**Conclusions:**

A total score that includes a combination of parameters such as LAD >3.785 cm, IVSd>1.165 cm, SV >79.5 ml, QTc >438.5 ms, and pericardial effusion can help describe the risk of new-onset PH.

## Introduction

Pulmonary hypertension (PH) is the most common complication and cause of death in patients with end-stage renal disease (ESRD) with a significantly higher incidence in patients with ESRD than in the general population. Persistent worsening of PH can lead to right heart failure and even death, which can be an independent disease, a complication, or a syndrome. PH significantly increases the mortality of patients with end-stage renal disease. As per the National Institutes of Health (NIH), United States, the natural median survival time of patients with PH is only 2.8 years, and it is also known as a cardiovascular “malignant tumor.” Clinical studies have shown that early treatment of PH can reduce the incidence of these complications. However, the clinical symptoms of patients with early-stage PH are atypical and relatively insidious. Hence, these patients are usually diagnosed after severe complications. These patients are diagnosed much later after the first symptom of PH, which is not conducive to early prevention and control and increases the medical burden (1). Therefore, early identification of patients with potential PH is critical for developing preventive strategies (2). Recent cohort studies have reported that 5%–15% of patients with ESRD develop PH, and early interventional PH-targeted therapy benefits patients with milder symptoms (3, 4). Despite some success in estimating mean pulmonary arterial pressure (mPAP) by echocardiography, its accuracy and determining the estimate remain challenging (5–7). Moreover, the addition of the S-wave and R-wave amplitudes of each lead of the electrocardiogram (ECG) to the PH risk algorithm may better identify PH risk. Nevertheless, whether a combination of clinical, ECG, and echocardiographic parameters can predict the development of PH in patients with ESRD remains to be elucidated. Herein, we evaluated the predictive value of the combined score, including these parameters, this study aimed to evaluate the ability of clinical, ECG, and echocardiographic parameters to predict the development of PH in patients with ESRD.

## Methods

Data from 370 patients with new-onset ESRD who underwent routine echocardiography and electrocardiography at the First Affiliated Hospital of Anhui Medical University between May 2016 and May 2017 were retrospectively reviewed. ESRD is diagnosed when the stimated Glomerular Filtration Rate (eGFR) falls below 15 ml/min/1.73 m^2^. In other words, when chronic kidney disease reaches stage 5, it enters ESRD. The inclusion criteria were:(a) diagnosed with ESRD in <3 months, (b) hemodialysis or no dialysis, and (c) echocardiography showed normal pulmonary artery pressure. The exclusion criteria were:(a) echocardiography has suggested pulmonary hypertension; (b) diagnosed with ESRD for >3 months; (c) already on peritoneal dialysis; (d) congenital heart disease, chronic pulmonary heart disease, Thyroid disorders, pulmonary stenosis, outflow tract obstruction, and interstitial lung disease; and (e) incomplete data records. Of the 370 patients, 21 were excluded due to incomplete echocardiographic data among other reasons; hence, the final analysis involved a total of 349 patients, all of whom were Han Chinese. The Ethics Committee of the First Affiliated Hospital of Anhui Medical University approved the study's protocol (Quick-PJ2022-13-18), and informed consent was waived. According to the guidelines for the diagnosis and treatment of PH, patients were initially screened according to the medical history of symptoms such as dyspnea, syncope, chest pain and other signs such as jugular venous filling and hepatic congestion. PH was suspected by ECG showing right heart enlargement, chest x-ray showing pulmonary vasodilatation, and echocardiography estimated that the peak velocity of tricuspid regurgitation was 2.9–3.4 m/s with other signs of PH, the pulmonary artery systolic pressure was 37–50 mmHg, or the peak velocity of tricuspid regurgitation was >3.4 m/s. Pulmonary artery systolic pressure >50 mmHg was used to determine a high likelihood of PH. Combined with clinical symptoms and signs and laboratory tests, 284 cases were considered to be caused by left heart disease, lung, hypoxia and other reasons, and the underlying diseases were mainly treated. 65 cases could not be determined by the above examination, and finally PH was diagnosed by the mean pulmonary artery pressure (mPAP) measured by right heart catheterization >25 mmHg.

### Data collection

General clinical data, such as name, sex, age, height, weight, smoking history, history of alcohol intake, underlying diseases, clinical biochemical indicators, and medical and family histories, were collected.

### ECG

Routine ECG examinations using a comprehensive electrocardiographic analyzer with a sensitivity of 10 mm/mV and a paper-travel speed of 25 mm/s were conducted for all patients. A 12-lead synchronous ECG was recorded in the supine position once a month. PR and QRS intervals were measured using leads V3 and V4 of the 12-lead ECG, and the QT interval was measured using lead II and V2. The corrected QT interval (QTc) was calculated using Bazett's formula. Abnormal ECG manifestations, such as right bundle branch block (RBBB), right axis deviation, right ventricular hypertrophy, and ST-T changes, were also recorded.

### Echocardiography

Echocardiography was performed for all patients using IE-33 and IE-Elite Color Doppler ultrasound monitors (Philips, USA). The S5-1 probe was used at a of 3–5 MHz. Transthoracic echocardiography is performed by an experienced sonographer. Ultrasound screening was performed on average every month. Measurements in standard four- and two-chamber views included aortic diameter (AO), left atrial diameter (LAD), pulmonary artery diameter (PA), interventricular septum thickness in end-diastole (IVSd), stroke volume (SV), left ventricular shortening rate (LVFS), left ventricular posterior wall thickness (LVPW), left ventricular end-diastolic volume (LVEDV), left ventricular end-systolic volume (LVESV), left ventricular end-diastolic diameter (LVDd), and left ventricular end-systolic diameter (LVDs). left ventricular ejection fraction (LVEF) was calculated by dividing the difference between LVEDV and LVESV by LVEDV. Fractional shortening (FS) was calculated using the following formula: (LVDd−LVDs)/LVDd. Left ventricular mass was calculated using the following formula: left ventricular (LV) mass = 1.05 × [(LVDd + LVPW + IVSd)^3^−LVDd^3^]−13.6 g and normalized to body surface area to obtain the LV mass index (LVMI).

Using pulsed-wave Doppler echocardiography, mitral flow velocity profiles were recorded in either the apical four-chamber view or the apical long-axis view, with the sampling volume at the mitral apex during diastole. Peak E (early diastolic) and A (late diastolic) wave velocities were measured. Moreover, the tricuspid regurgitation pressure gradient (TRPG) was also measured.

### During follow-up

Data on new-onset PH were obtained during follow-up hospital examinations, and the incidences of new-onset PH were analyzed.

### Statical analysis

The parameters for continuous data that were normally distributed and expressed as mean ± standard deviation and those that had a skewed distribution were expressed as median (M) and interquartile range. Countable data were expressed as percentages. All statistical tests were two-sided, and a *P* value of <0.05 was considered statistically significant. Measurement data were analyzed using the Mann–Whitney *U*-test or *t*-test. Pearson's and *χ*^2^ tests were used for analyzing countable data to compare the clinical characteristics and measure the risk factors of patients with and without new PH. From this analysis, potential predictors of PH were identified, and continuous independent variables with *P* < 0.05 were analyzed using receiver operating characteristic (ROC) curves to determine their optimal cut-off values. The best cut-off point for each marker was obtained using the Youden index. According to the optimal cut-off value from the ROC curve analysis, univariate and multivariate Cox proportional hazards models were input to determine the influence of various risk factors on new-onset PH during the follow-up period. In the Cox model, Kaplan–Meier curves for no PH events were calculated to describe the occurrence of new PH events by risk factors. Differences between high and low levels of these factors were tested using the log-rank test.

## Result

Patients who did not meet the inclusion criteria and those with incomplete data records were excluded, and finally, 349 patients (mean age 35 ± 8 years, 72.0% male) were included in the final analysis.

### Clinical features

Table 1 summarizes the clinical characteristics of patients according to new-onset PH status. No significant differences in age, sex, body mass index (BMI), smoking history, hemoglobin, albumin, hypertension, hyperlipidemia, diabetes, coronary heart disease, and cardiomyopathy were noted between patients with and without PH.

**Table 1:** Subject characteristics vs. PH Status.

Variable	No PH (191)	PH (158)	*P*
Age (years)	34.9 ± 8.4	35.8 ± 7.6	0.295
Male (%)	136 (71)	116 (73)	0.646
BMI (kg/m2),median (Q1-Q3)	21 (18.8–23.5)	21.6 (19.3–24.6)	0.088
Hypertension	163 (85)	131 (83)	0.535
Diabetes mellitus	32 (17)	24 (15)	0.692
Dyslipidemia	29 (15)	27 (17)	0.629
CAD	81 (42)	74 (47)	0.407
Cardiomyopathy	41 (21)	32 (20)	0.782
HF	100 (52)	82 (52)	0.932
History of smoking	53 (28)	40 (25)	0.609
Haemoglobing/L, median (Q1-Q3)	110 (86–119)	114 (93–121)	0.052
Albuming/L, median (Q1-Q3)	38 (31–42)	41 (35–45)	0.077

Continuous data are presented as mean ± standard deviation or median (interquartile range). Categorical data are presented as numbers and percentages. PH, pulmonary hypertension; BMI, body mass index; CAD, coronary artery disease; HF, heart failure.

### Baseline characteristics

Patients with new-onset PH had higher QTc intervals and longer maximal QTc intervals on ECG compared to patients without PH (Table 2). Moreover, compared to patients without PH, those with PH had a longer QT interval and higher R5 voltage. Contrarily, no significant differences in P wave time, heart rate, PR interval, QRS axis, left and right bundle branch block, prior myocardial ischemia, or atrial fibrillation were present between patients with and without PH.

**Table 2:** Electrocardiography vs. PH Status.

Parameter	No PH (191)	PH (158)	*P*
P wave duration (ms)	96 (88–102)	98 (92–104)	0.078
Heart rate (bpm)	78 (70–89)	78 (71–87)	0.806
PR interval (ms)	152 (140–166)	152 (138–164)	0.812
QT time (ms)	368 (344–392)	376 (354–396)	0.038
QTc time (ms)	425 (407–438)	430 (414–446)	0.016
QRS interval (ms)	92 (84–98)	92 (88–100)	0.282
QRS axis (degrees)	100 (49–264)	208 (169–249)	0.068
R/S-wave voltages (S1 + R5) (mv)	2.95 (2.4–3.59)	3.13 (2.59–3.97)	0.038
R5-wave voltages (mv)	1.64 (1.31–2.16)	1.93 (1.50–2.58)	0.001
S1-wave voltages (mv)	1.15 (0.8–1.53)	1.24 (0.85–1.65)	0.313
ST-segment depression	41 (21)	30 (19)	0.567
Left bundle branch block *n* (%)	17 (9)	14 (9)	0.990
Right bundle branch block *n* (%)	13 (7)	9 (6)	0.671
Previous MI *n* (%)	17 (9)	15 (10)	0.834
Atrial fibrillation *n* (%)	18 (9)	13 (8)	0.709

Data are given as *n* (%) or median (interquartile range); PH, pulmonary hypertension; QT, QT interval; QTc, corrected QT interval.

The echocardiographic LAD, LVDd, IVSd, SV, and LVPW of patients with new-onset PH were significantly higher than those without PH. Furthermore, the probability of pericardial effusion and left ventricular hypertrophy was significantly higher in those with new-onset PH than in those without PH. However, the AO, ratio of early-to-late diastolic filling peak-flow velocities (E/A ratio), or the reduction of left ventricular compliance did not affect the incidence of new-onset PH. (Table 3).

**Table 3:** Echocardiography vs. PH Status.

Parameter	No PH (191)	PH (158)	*P*
AO (cm)	3.15 ± 0.25	3.19 ± 0.26	0.107
LAD (mean ± SD) (cm)	3.58 ± 0.47	4.13 ± 0.62	<0.001
LVDd (cm) mean ± SD	4.93 ± 0.48	5.40 ± 0.64	<0.001
IVSd (cm)	1.08 (0.99–1.19)	1.18 (1.06–1.23)	<0.001
SV (ml)	73 (62–79)	84 (72–99)	<0.001
LVPW (cm)	1.02 (0.94–1.14)	1.07 (0.98–1.15)	0.004
LVEF (mean ± SD)	63 (61–65)	61 (57–65)	0.001
FS (%)	34 (33–36)	33 (30–36	0.011
E/A	135 (71)	125 (79)	0.072
LVH	89 (47)	128 (81)	<0.001
pericardial effusion	23 (12)	64 (41)	<0.001
Decreased left ventricular compliance	123 (64)	112 (71)	0.198

Data is presented as mean ± SD, *n* (%), or median (interquartile range). AO, aortic diameter; LAD, left atrial diameter; LVDd, left ventricular end-diastolic dimension; IVSd, Interventricular septum thickness in end-diastole; SV, stroke volume; LVPW, left ventricular posterior wall thickness; LVEF, left ventricular ejection fraction; FS, fractional shortening; E/A, ratio of early-to-late diastolic filling peak-flow velocities; LVH, Left ventricular hypertrophy.

### Prediction of new PH

**Figure 1:**
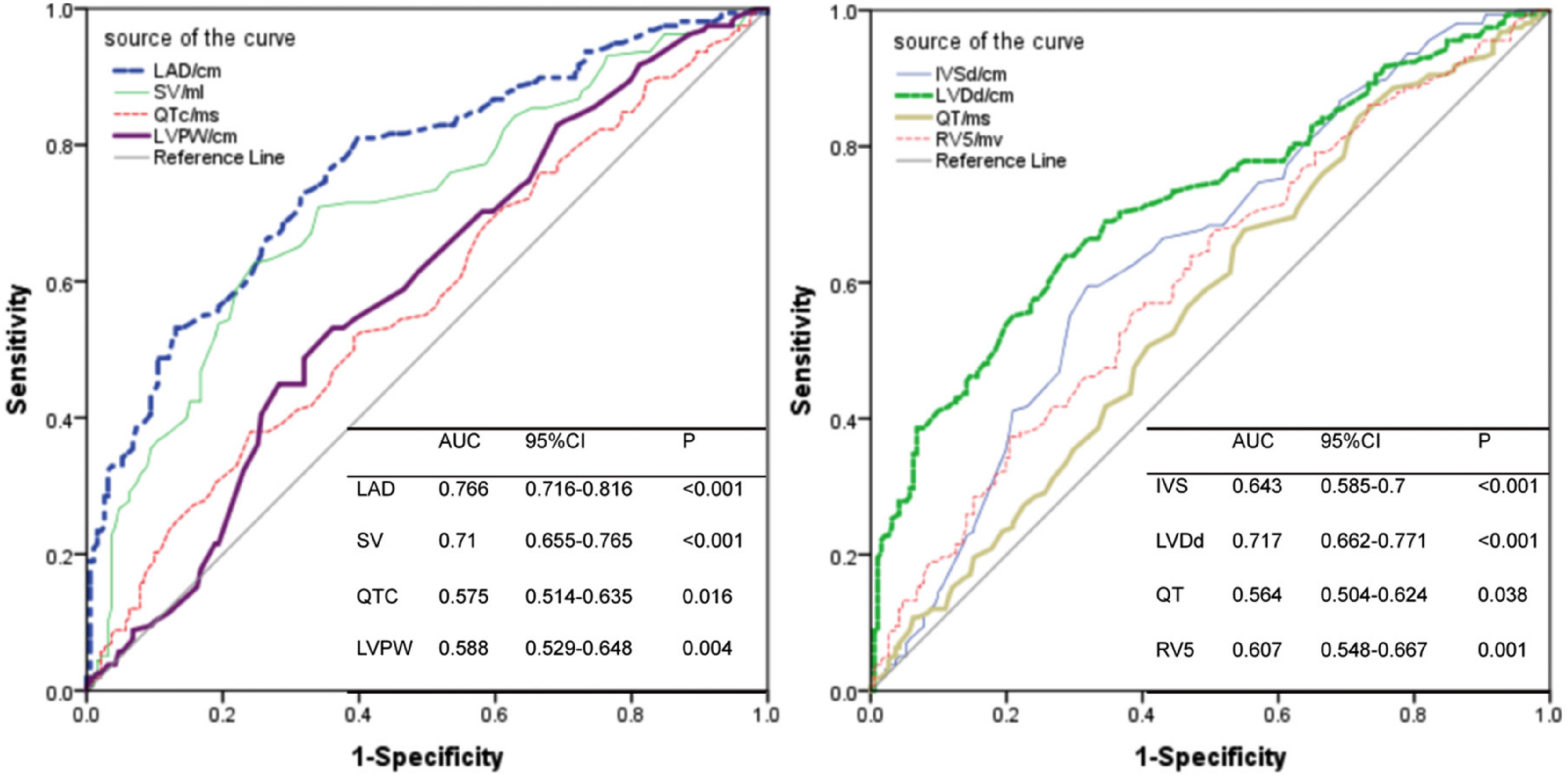
Receiver operating characteristic curve revealing the area under the curves for LAD, SV, QTc, LVPW, IVSd, LVDd, QT and RV5 to be associated with the presence of PH (AUC area under curve, CI confidence interval). In the Cox models, for continuous independent variables, receiver operating characteristic (ROC) curve analysis was used to determine optimal cut-offs of continuous variables. Optimal cut-off points were obtained for each marker using the Youden index. Kaplan–Meier curves for freedom from PH events were calculated to describe the occurrence of PH events by risk factor based on the best cut-off on ROC curve analysis.

**Figure 2:**
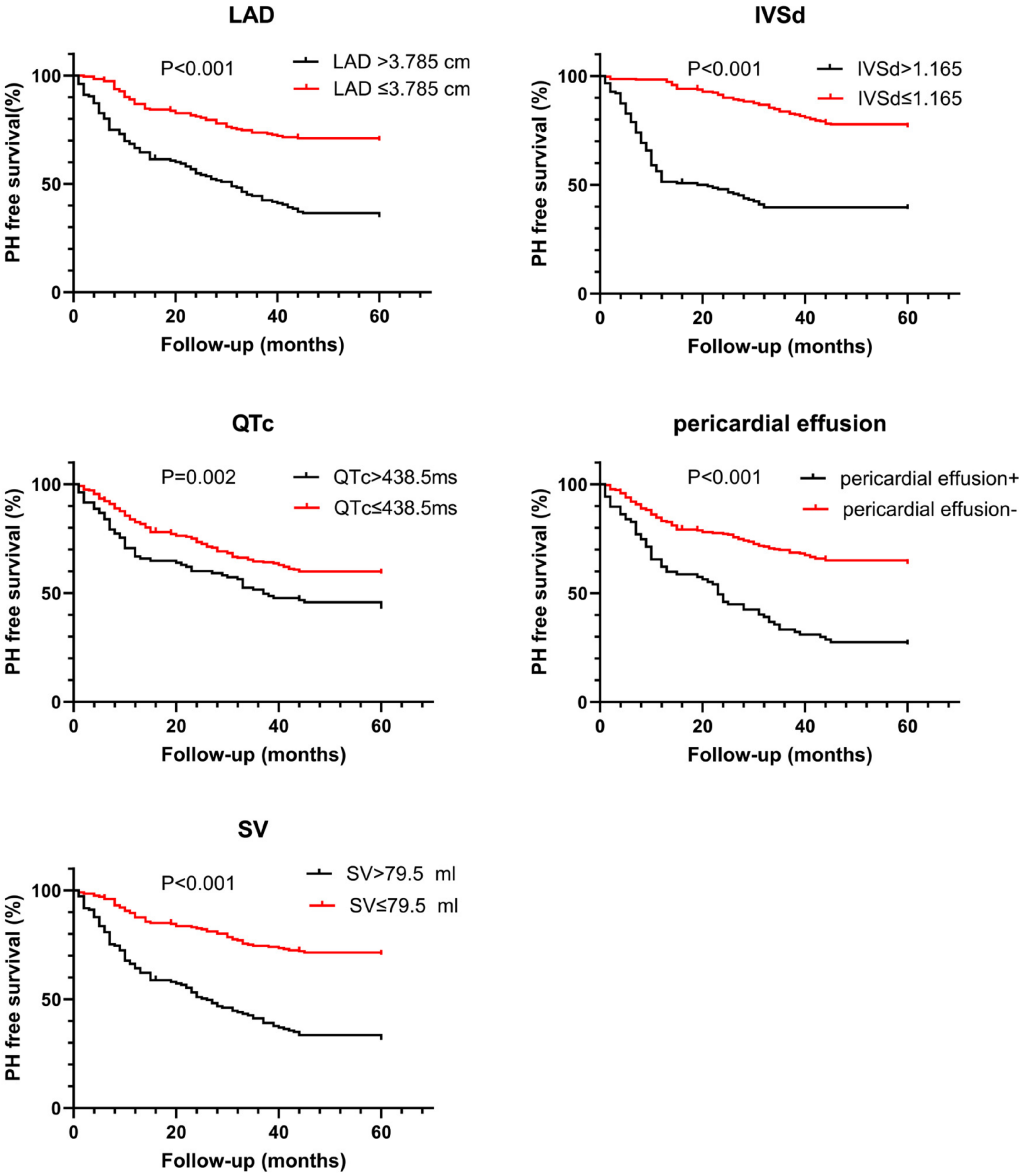
Kaplan–Meier estimates of survival free from PH according to left atrial diameter (LAD>3.785 cm), interventricular septum thickness in end-diastole (IVSd>1.165 cm), stroke volume (SV>79.5 ml), corrected QT interval (QTc>438.5 ms), and hydropericardium.

**Figure 3:**
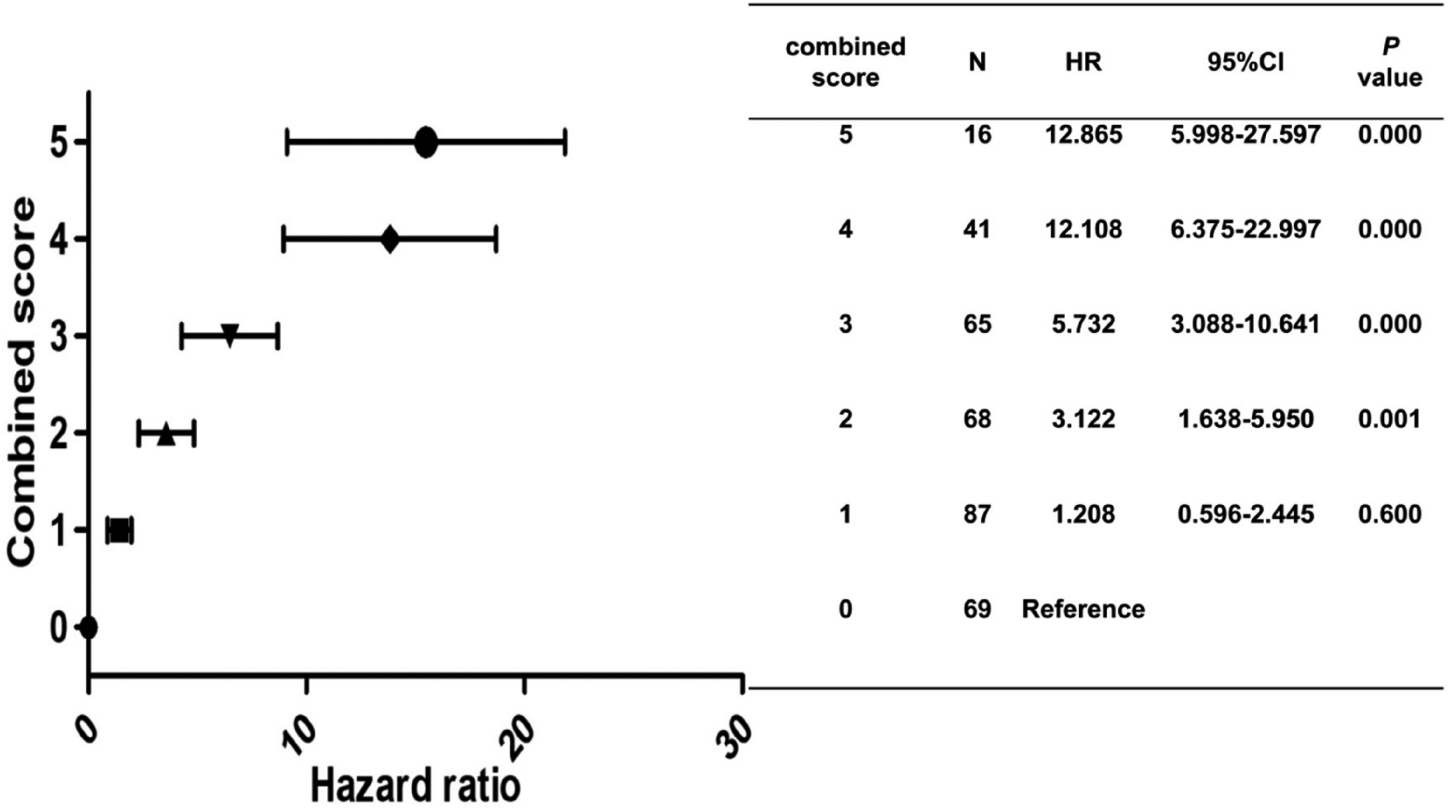
Hazard ratio for PH according to the combined score.

**Figure 4:**
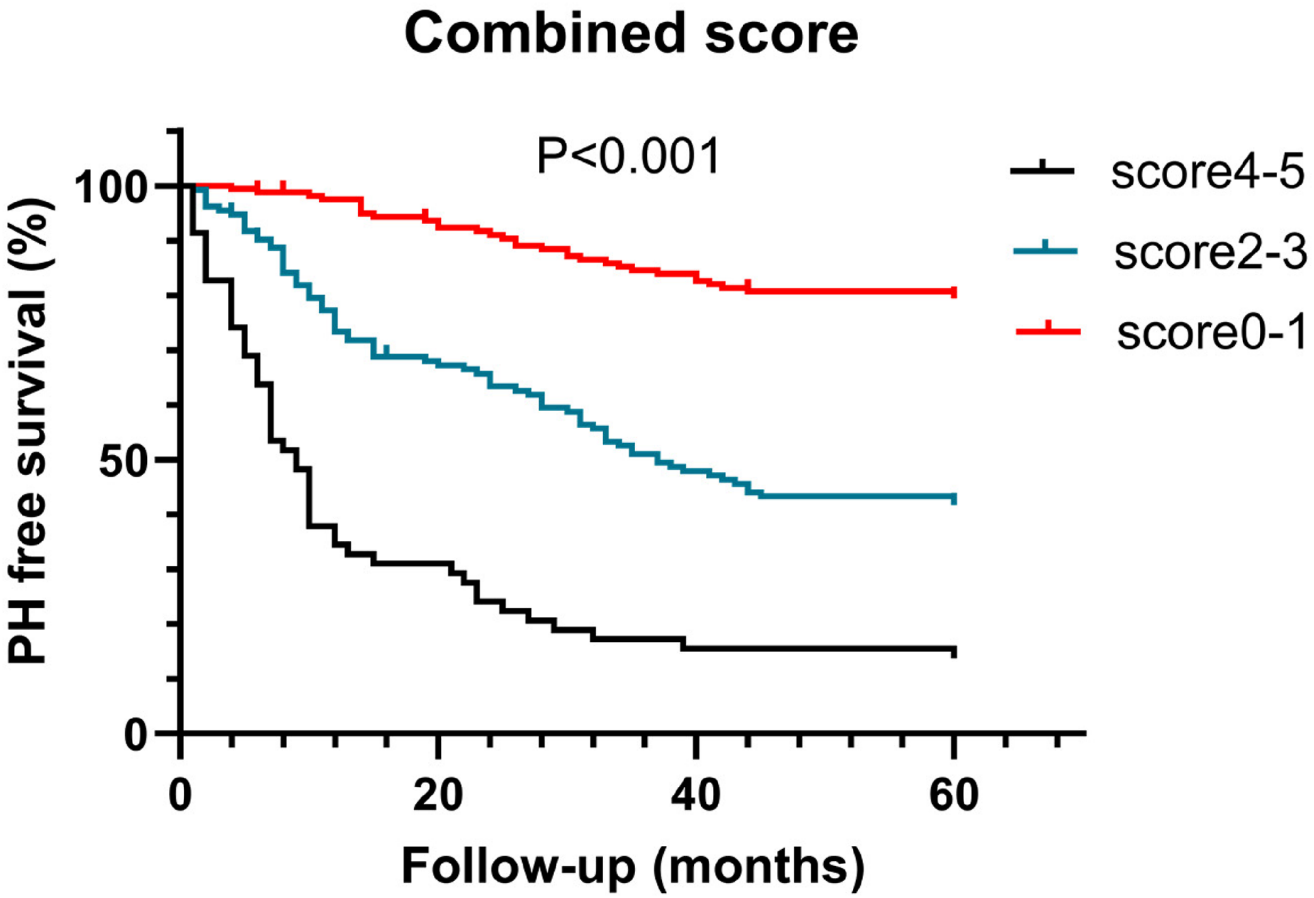
Kaplan–Meier estimate of survival free from new-onset PH according to the combined score for new-onset PH.

Statistically significant variables associated with new-onset PH (Tables 1–3) were entered into a univariate Cox proportional hazards model (Table 4). For Cox ratio analysis and Kaplan–Meier curves without PH, cut-off values for possible risk factors were determined based on ROC curve analysis (Figure 1). Ejection fraction (EF) and FS were excluded from the stepwise selection-based multivariate analysis. In the multivariate Cox analysis, LAD, IVSd, SV, QTc, and pericardial effusion were found to be independently associated with the development of PH [multivariate-adjusted hazard ratio (HR)]: LAD >3.785 cm, 2.256; IVSd>1.165 cm, 2.624; SV>79.5 ml, 2.618; QTc>438.5 ms, 1.844; and pericardial effusion, 2.127; Table 4). The results of the Kaplan–Meier analysis for freedom from PH indicated that the risk of a new occurrence of PH was higher in patients with longer LADs (>3.785 cm), longer IVSds (>1.165 cm), more SV (>79.5 ml), longer QTc (>438.5 ms), and more pericardial effusion than in patients without PH (Figure 2). A significant increase was seen in the HR for new-onset PH composite scores of 3, 4, or 5 (composite score 3, HR, 5.732; 95% CI: 3.088–10.641, *P* < 0.001; score 4, HR, 12.108; 95% CI: 6.375–22.997, *P* < 0.001; score 5, HR, 12.865; 95% CI: 5.998–27.597, *P* < 0.001) (Figure 3). Patients were divided into three risk groups for PH based on their composite scores: scores 0–1, 2–3, and 4–5). In the Kaplan–Meier curve analysis, the PH incidence increased significantly with increasing composite scores (Figure 4). The combined score area under the curve (AUC) based on these five parameters was 0.785.

**Table 4:** Independent indicators of PH.

	Unadjusted (349)			Adjusted (349)		
	HR	95% CI	*P* -value	HR	95% CI	*P*-value
LAD>3.785 (cm)	3.974	2.794–5.651	<0.001	2.256	1.367–3.722	0.001
IVSd>1.165 (cm)	2.758	2.008–3.787	<0.001	2.624	1.512–4.556	0.001
LVDd>5.135 (cm)	2.964	2.14–4.105	<0.001	0.991	0.533–1.844	0.977
SV>79.5 (ml)	3.298	2.385–4.561	<0.001	2.618	1.443–4.750	0.002
FS<36.5	0.939	0.624–1.412	0.761	0.901	0.336–2.414	0.836
EF<66.5	0.897	0.576–1.396	0.630	0.634	0.210–1.917	0.419
LVPW>1.065 (cm)	2.128	1.555–2.911	<0.001	0.836	0.508–1.377	0.482
QT>363 (ms)	1.57	1.125–2.193	0.008	1.099	0.725–1.664	0.656
QTc>438.5 (ms)	1.63	1.182–2.248	0.003	1.844	1.169–2.909	0.008
LVH	3.909	2.623–5.826	<0.001	1.356	0.728–2.524	0.337
pericardial effusion	2.83	2.055–3.896	<0.001	2.127	1.400–3.234	<0.001
E/A<1	1.458	0.994–2.140	0.054	0.906	0.429–1.913	0.795
R5-wave voltages (mv)>1.825	1.754	1.281–2.402	<0.001	1.453	0.942–2.241	0.091

PH, pulmonary hypertension; LAD, left atrial diameter; IVSd, Interventricular septum thickness in end-diastole; LVDd, left ventricular end-diastolic dimension; SV, stroke volume; FS, fractional shortening; EF, ejection fraction; LVPW, left ventricular posterior wall thickness; QT, QT Interval; QTc, corrected QT interval; LVH, Left ventricular hypertrophy; E/A, ratio of early-to-late diastolic filling peak-flow velocities.

## Discussion

This is the first study to establish a comprehensive scoring model for predicting the PH occurrence in patients with ESRD using ECG, ultrasonography, and clinical factors. This study showed that LAD, IVSd, SV, QTc, and pericardial effusion were independently associated with an increased risk of developing new-onset PH. PH increases the risk of dyspnea, heart failure, lung inflammation, cardiac enlargement and hypertrophy, tricuspid valve insufficiency, and atrial fibrillation. Early recognition of PH, although difficult to perform clinically, can affect the occurrence of these clinical events. Conventionally, right heart catheterization is used to accurately diagnose pulmonary arterial pressure; however, it is not widely popular because of its associated trauma. Identifying PH by clinical symptoms and echocardiography has a noticeable lag. Sometimes, PH is not correctly identified, thereby increasing the incidence of cardiovascular and cerebrovascular accidents in patients with ESRD. Hence, methods to detect PH early in its latent period in such patients are crucial along with intervention to reduce the occurrence of clinical events.

In the present study, BMI, hyperlipidemia, hypertension, diabetes, and coronary heart disease were not associated with developing PH. However, Nakhoul et al. found that the incidence of PH in patients with diabetes was significantly higher than in healthy individuals (8). Etemadi et al. found that hemoglobin, albumin, and serum iron levels differed in patients with PH and those with normal pulmonary arterial pressure in patients undergoing maintenance hemodialysis (9). Small sample sizes and heterogeneity in disease duration and treatment may have contributed to the discrepancies between the results of the current and previous studies. In the present study, BMI was also not associated with new-onset PH. However, Juan et al. reported that excessive weight gain during the interdialytic period and increased cardiac and circulatory volume load can lead to pulmonary vascular remodeling (10). As the vessel membrane is significantly thickened, the pulmonary circulatory resistance increases and so does the pulmonary arterial pressure. This difference may be attributed to the inconsistency of the comparison objects. The former uses the body weight of the sample individuals before and after the dialysis period as a reference rather than the comparison between the study individuals, as performed in the present study.

In the present study, the ECG parameters, namely QT time, QTc time, R5, and S1 voltage, were associated with PH. Consistent with the results of the studies by Sato S and Waligóra M, we also found that ECG is a reliable method for PH diagnosis, treatment response, and survival prediction (11, 12). Bouchery-Barde et al. analyzed the risk factors, such as ECG parameters, in patients with PH (13). They reported that prolongation of the QT interval increases the risk of death, reflecting abnormal ventricular conduction and remodeling. Framingham et al. also reported that the QRS interval positively correlates with left ventricular mass, end-diastolic diameter, interventricular septum, and left ventricular posterior wall thickness (14). Thus, the prolongation of the QRS interval indicates a decrease in cardiac function. With the prolongation of the QRS interval, its sensitivity for predicting the decrease in cardiac function gradually increases. Furthermore, Sun P-Y et al. also demonstrated the importance of QRS prolongation as a predictor of mortality (15). They found that patients with idiopathic PH with a QRS duration of ≥0.12 s had a 2.5-fold higher risk of death than those with a normal QRS duration. In this study, prolonged QT time, prolonged QTc time, and high S1 voltage were predictive of PH. Since occult PH may induce ventricular nodal dysfunction, further detailed studies are needed.

In the present study, the echocardiographic parameter associated with PH was LAD. This is consistent with the results of the Agarwal et al. (16). The structure and function of the left heart are suggested to affect PH occurrence. Simultaneously, an increase in pulmonary arterial pressure may further aggravate the cardiac pathology in patients undergoing maintenance hemodialysis (17). Consistent with the results of previous studies, we also found that SV has the highest clinical predictive value for new-onset PH (18). PH in patients undergoing maintenance hemodialysis is associated with increased cardiac output and cardiac index (19). Increased cardiac output leads to an increase in the pulmonary arterial pressure. The mechanism may activate the vascular endothelial system, increase vascular endothelin, thicken the intima of large and medium arteries, and further increase the cardiovascular load (20). These studies showed that certain echocardiographic parameters, including increased LAD, decreased echocardiographic surrogate for pulmonary arterial capacitance, increased left intraventricular diameter wall thickness, and decreased left intraventricular systolic function, predict the risk of PH (21). For example, hypertension or hypertensive heart disease has been shown to contribute to the development of left atrial (LA) enlargement.

Contrarily, normal aging does not lead to an increase in LA. Eric R et al. confirmed that pericardial effusion is extremely common in PH, which is consistent with the results of the present study (22). The presence and extent of pericardial effusion are related to the mortality of PH. Even moderate pericardial effusion is associated with significantly increased mortality, and the presence of pericardial effusion implies an increased risk of subsequent mortality (23). Similarly, Krämer et al. described that the carbon dioxide production slope (VE/VCO2 slope) assessed by cardiopulmonary exercise testing, right ventricle Sokolow–Lyon index, and tricuspid regurgitation pressure gradient were closely related to the severity of idiopathic PH (24, 25). In the present study, echocardiographic parameters other than LAD, including FS, LVMI, LVEDV, LVEF, and E/A ratio, were not associated with PH, which was a finding that was inconsistent with the results of previous studies. Considering the group of patients with ESRD in this study, the primary renal diseases include hypertension, diabetes, and chronic nephritis all of which require hemodialysis treatment, which specifically interferes with the cardiovascular system; these patient characteristics may cause the findings of this study to be closely related to the disease and may account for the differences between our results and those of previous studies.

In the present study, a composite score was used for PH prediction, which was calculated as a weighted score (LAD>3.785 cm, 1; IVSd>1.165 cm, 1; SV>79.5 ml, 1; QTc>438.5 ms, 1; pericardial volume liquid, 1). The study found a significant increase in HR for PH with a combined score of 3, 4, and 5 compared with 0. This is consistent with the current finding that these five parameters are independently associated with PH development. There are currently no studies evaluating the combined predictive power of clinical, ECG, and echocardiographic parameters for PH. Nevertheless, some studies suggest the use of clinical, ECG, or clinical and echocardiographic parameters combined or clinical, laboratory, and usefulness of echocardiographic parameter combinations.

The AUC of the current composite score (0.785) is similar to previous risk stratification scores, such as COMPERA or REVEAL 2.0, and the addition of parameters can improve its diagnostic and prognostic accuracy (26, 27). Nevertheless, these scoring systems contain more parameters than those used in the present study and are extremely complex for clinical use. Follow-up studies with adequately sized study groups and a wide range of disease severities are needed to confirm the validity and usefulness of our scoring system. Mańczak R et al. found that a multivariate logistic regression model with three echocardiographic parameters, including inferior vena cava inspiratory diameter (IVCin), right atrium area (RAA), tricuspid annulus plane systolic excursion (TAPSE), and one biochemical marker pro-Brain Natriuretic Peptide (NTproBNP) was superior to any tested univariate model and more accurate in identifying hemodynamic characteristics of patients with PH (28). Sehgal S and other studies measured the time spent at different activity levels and steps per day as an alternative tool to monitor the severity of the disease (29). Reduced physical activity may be a potential biomarker of clinical deterioration. Since the current parameters can be easily evaluated in any clinic or hospital, this scoring system, which has fewer parameters than other scoring systems, can be easily applied clinically. Moreover, timely interventions to prevent complications, including right heart hypertrophy, dilation, and heart failure, can be performed in patients in whom the risk of PH is identified.

In the present study, Kaplan–Meier estimation of the prediction of PH based on the total score or each parameter shows that the difference in the incidence rate of PH increases after 30 months of follow-up. This indicates that the total score can predict the occurrence of postoperative PH≥4–5 years. In Zaky A et al.'s study (a community-based cohort study), different types of ECG, biochemical, and echocardiographic parameters were determined through a series of measurements (30). These parameters can be used for early detection of PH, monitoring the progress of PH, and evaluating right ventricular dysfunction and its treatment response. M Tudoran et al. found that PH was associated with increased pulmonary vascular resistance, prolonged hemodialysis duration, and chronic glomerulonephritis in ESRD patients undergoing hemodialysis (31). Fadaii A et al. showed that pulmonary hypertension was associated with dialysis duration, age and ejection fraction (32). Compared to their study, the present study used a composite score to screen for new-onset PH in patients with ESRD with a median follow-up period of 30 months.

### Limitations

First, this study is a retrospective, single-center study with a relatively small sample size. Further prospective studies with larger sample sizes on more patients to determine their clinical significance. Secondly, the incidence of mild PH may be underestimated because some asymptomatic patients may not be diagnosed with PH. Third, right heart catheterization was not universally available. The main reason was that the invasive examination was too expensive, and the patients rejected it. Instead, pulmonary artery pressure was evaluated by echocardiography, which had certain limitations. Fourth is the potential population selection bias because all patients were diagnosed with ESRD in a relatively short time, and dialysis was performed via hemodialysis; hence, the patients could be followed up regularly.

## Conclusions

In conclusion, the new risk of PH in patients with ESRD should be evaluated by comprehensive parameters, such as ECG and echocardiography. The higher the comprehensive score of LAD >3.785 cm, IVSd >1.165 cm, SV >79.5 ml, QTc >438.5 ms, and pericardial effusion, the higher the probability of PH.

## Data Availability

We want to thank all the teachers in the Urology Department of the First Affiliated Hospital of Anhui Medical University for their excellent technical assistance and superb statistical help.
